# Delivering cancer services: a multi-disciplinary approach

**DOI:** 10.2349/biij.2.2.e31

**Published:** 2006-04-01

**Authors:** LM Tho, DWY Wong

**Affiliations:** 1 Cell-Cycle and Checkpoint Laboratory, Beatson Institute for Cancer Research, Glasgow, United Kingdom; 2 Department of Clinical Oncology, Birmingham Cancer Centre, Birmingham, United Kingdom

**DEAR SIR,**

The article by Lim [1] on the development of oncology services in Malaysia was both insightful and comprehensive. It is interesting to read that cancer services are growing from strength to strength, with a national cancer institute planned. The field of oncology has been transformed over the last few decades with a proliferation in technological advances and a revolution occurring in molecular medicine. Interestingly, the thinking surrounding cancer service delivery has also been changing. Cancer care is increasingly being delivered within a multidisciplinary team environment, involving a host of highly skilled professionals. As oncologists, despite our unique skills in the diagnosis and treatment of cancer [2] we are, but a cog (albeit a necessary one), in a big wheel that is required to manage this complex disease. Therefore, it is arguable that oncology might actually be considered a multi-disciplinary specialty.

Every oncology department relies on a team of highly trained radiographers, physicists, pharmacists, nurses, and support staff for everyday functioning. With the resurgence of radiation research and development, intensity modulated and image guided radiotherapy [3] being prime examples, our reliance on our physicist and radiographer colleagues has never been greater. This includes all aspects of radiotherapy delivery, machine commissioning, quality assurance, treatment planning, and research. It has also been recognized that cancer centres benefit from taking an active role in public education and outreach, as this often leads to drastic improvements in patient satisfaction and overall perceptions.

The concept of teamwork extends far beyond our own departments. One of our inseparable partners is radiology, which has evolved into a vast and multifaceted discipline. Different forms of imaging are used during a patient’s clinical course to diagnose, stage, plan, deliver intervention, and detect recurrence. Standard workhorses such as plain radiography and computed tomography (CT) are invaluable, but more specialized imaging is also important, including magnetic resonance imaging for detecting spinal cord lesions or imaging pelvic anatomy, bone scans for detecting skeletal metastases, and radiofrequency ablation for treating liver metastases. Even the most subtle of radiological features may predict a patient’s outcome, for example, the presence of rectal tumour found within 1 mm of the mesorectal fascia on a T2 weighted MRI scan could signify a substantial increase in the chance of local recurrence and warrant aggressive downstaging by preoperative chemoradiotherapy [4].

An emergent technology is CT-positron emission tomography (CT-PET). It offers undeniably superior imaging quality and evidence of its efficacy is emerging for various tumour sites [5,6]. In addition, it is creating vast opportunities for in vivo imaging research, which is revolutionising the way drug trials are being designed (e.g., non-invasive pharmacokinetic and pharmacodynamic studies [7]) and the way molecular research is conducted. These benefits must be balanced by the cost of providing this service. Not only does it incur an initial set-up cost of at least £4 million/MYR 25.5 million (scanner and cyclotron) and annual running costs of at least £1.2 million/MYR 7.5 million [8], but requires the support of specialist radiopharmacists, physicists, and radiologists. Clearly, the best value for money would be for a multidisciplinary team to fully utilise this technology.

Another specialty that we work closely with is pathology. Good pathological examination enables the right diagnosis to be made and, consequently, the right treatment to be delivered. This is especially critical when dealing with curable conditions. For each tumour type, different tumour characteristics can serve as either prognostic factors (to predict disease behaviour, e.g., recurrence rates, overall survival) or predictive factors (to predict tumour responses to anti-cancer therapy). For example, in breast cancer, the presence of lymph node involvement, lymphovascular space invasion, and a high tumour grade confers a poor prognosis, while hormone receptor or HER-2 receptor status would predict for a response to anti-oestrogen therapy or trastuzumab (Herceptin), respectively [9]. Such routine analysis often requires the support of highly specialised facilities and staff. Furthermore, new techniques are constantly being developed, eg., multi-gene and multi-protein analysis using gene-array and protein-array platforms [10], and this requires continued collaboration to evaluate and apply these technologies appropriately.

Another key player in oncology is undoubtedly the surgeon. Modern surgical oncology practices, for example, total mesorectal resection in rectal cancer, maximal debulking in ovarian cancer, and nephrectomies in renal cancers have radically improved survival outcomes. The correct interplay between chemotherapy, radiotherapy, and surgery is critical, and one of the best ways of ensuring optimal sequencing and minimising delays is to build close working partnerships amongst professionals in these specialties. In the general care of the cancer patient, clinical oncologists often rely on input from their fellow specialists. This can be in the form of support of the critically ill patient (intensive care/anaesthetists), management of malignancy induced surgical complications, e.g., bowel perforation or obstruction (surgeons), or stabilisation of pathological fractures (orthopaedics), managing infectious or other medical complications (physicians), blood product support for patients undergoing chemotherapy (blood bank/haematologists), and pain management and end-of life care (palliative care/hospice).

The final area that relies on collaborative effort is oncology research. Clinical trial units rely heavily on the support of research nurses, data managers, and statisticians. Translational research and drug development requires close cooperation between clinicians and scientists and, increasingly, from industry. Good research tends to flourish where a critical mass of people are able to generate ideas and lend expertise. Many cancer centres have realised this and have sought to provide closer interactions between specialties by developing joint clinics and multidisciplinary meetings and seminars. For the various specialties involved, a degree of sub-specialization is required to ensure familiarity with the specifics of oncological practice. This can sometimes require housing cancer treatment centres, research institutes, and regional teaching hospitals in close physical proximity to one another. National initiatives have also recognised the need for a multidisciplinary approach. Within the world-famous US National Cancer Institute (NCI), designated cancer centres are “encouraged to stimulate collaborative research involving more than one field of study” [11]. In a visionary move, the NCI has established the Cancer Biomedical Informatics Grid [12], which aims to enable global communication and resource sharing throughout it vast network of centres. The nascent UK equivalent, the National Cancer Research Institute (NCRI) Informatics Initiative, is similarly promoting the integration of basic science and clinical activity [13].

It is clear that a multidisciplinary approach in treating cancer patients facilitates improvements in patient care and outcomes. Therefore, it is vital that we continue to forge strong links with colleagues from all specialties, particularly when faced with increasing complexities in the treatment of this challenging disease.
